# Preserved VPS13A distribution and expression in Huntington’s disease: divergent mechanisms of action for similar movement disorders?

**DOI:** 10.3389/fnins.2024.1394478

**Published:** 2024-06-05

**Authors:** Esther García-García, Maria Carreras-Caballé, Albert Coll-Manzano, Alba Ramón-Lainez, Gisela Besa-Selva, Esther Pérez-Navarro, Cristina Malagelada, Jordi Alberch, Mercè Masana, Manuel J. Rodríguez

**Affiliations:** ^1^Department of Biomedical Sciences, School of Medicine and Health Sciences, Institute of Neurosciences, Universitat de Barcelona, Barcelona, Spain; ^2^August Pi i Sunyer Biomedical Research Institute (IDIBAPS), Barcelona, Spain; ^3^Centro de Investigación Biomédica en Red de Enfermedades Neurodegenerativas (CIBERNED), Instituto de Salud Carlos III, Barcelona, Spain; ^4^Production and Validation Center of Advanced Therapies (Creatio), Faculty of Medicine and Health Science, University of Barcelona, Barcelona, Spain

**Keywords:** chorea-acanthocytosis, VPS13A, huntingtin, Huntington’s disease, movement disorders, basal ganglia, neurodegeneration

## Abstract

VPS13A disease and Huntington’s disease (HD) are two basal ganglia disorders that may be difficult to distinguish clinically because they have similar symptoms, neuropathological features, and cellular dysfunctions with selective degeneration of the medium spiny neurons of the striatum. However, their etiology is different. VPS13A disease is caused by a mutation in the VPS13A gene leading to a lack of protein in the cells, while HD is due to an expansion of CAG repeat in the huntingtin (Htt) gene, leading to aberrant accumulation of mutant Htt. Considering the similarities of both diseases regarding the selective degeneration of striatal medium spiny neurons, the involvement of VPS13A in the molecular mechanisms of HD pathophysiology cannot be discarded. We analyzed the VPS13A distribution in the striatum, cortex, hippocampus, and cerebellum of a transgenic mouse model of HD. We also quantified the VPS13A levels in the human cortex and putamen nucleus; and compared data on mutant Htt-induced changes in VPS13A expression from differential expression datasets. We found that VPS13A brain distribution or expression was unaltered in most situations with a decrease in the putamen of HD patients and small mRNA changes in the striatum and cerebellum of HD mice. We concluded that the selective susceptibility of the striatum in VPS13A disease and HD may be a consequence of disturbances in different cellular processes with convergent molecular mechanisms already to be elucidated.

## 1 Introduction

Basal ganglia disorders are a heterogeneous group of neurodegenerative diseases that affect selectively a group of neuronal populations in cortical and subcortical circuitries. VPS13A disease (chorea-acanthocytosis, ChAc, OMIM: 200150; Jung et al., 2011; Peikert et al., 2023) and Huntington’s disease (HD, OMIM: 143100; Ross et al., 2014) are two basal ganglia disorders that are often clinically difficult to distinguish because they have similar symptoms, magnetic resonance imaging findings (Suzuki et al., 2020) and neuropathological features with selective degeneration of the medium spiny neurons (MSNs) in the striatum (Liu et al., 2019). However, the etiology of both diseases is different. ChAc is a rare autosomal recessive neurodegenerative disease caused by a mutation in the VPS13A gene located on chromosome 9, which encodes the protein VPS13A (Ueno et al., 2001). Meanwhile, HD is an autosomal dominant disease that is due to an expansion of CAG repeat in the huntingtin (Htt) gene located on chromosome 4 (Ross et al., 2014). Thus, MSNs have a selective vulnerability to the lack of VPS13A (Henkel et al., 2008; Walterfang et al., 2011) and to the accumulation of mutant Htt (mHtt; Vonsattel and DiFiglia, 1998; Kassubek et al., 2004), while other neuronal populations in the striatum, such as interneurons, are more resistant. Therefore, the understanding of the mechanisms that make MSNs more vulnerable can be useful in developing therapeutical strategies to protect these neurons and their circuitries.

The low frequency of ChAc patients determines the little knowledge so far about the localization and function of VPS13A. This is a large ubiquitous protein highly expressed in the brain with a distinct VPS13A distribution that contributes to explaining the ChAc neuropathology (García-García et al., 2021). However, although the main neuropathological feature in ChAc patients is the selective degeneration of the caudate nucleus and putamen, the concentration of VPS13A in basal ganglia nuclei is weak (Kurano et al., 2007; García-García et al., 2021). Thus, the vulnerability of striatal neurons to VPS13A disease seems not to be related to the amount of protein present in the cell, but to specific striatal functional properties, circuitry, and MSN cell processes specifically affected by the lack of VPS13A.

VPS13A has a large variety of cellular functions that can affect the selective vulnerability of distinct neuronal populations. At the cellular level, VPS13A is a lipid transport protein localized in the contact sites between organelles (Kumar et al., 2018). Its lack of function has been associated with endocytic trafficking and lysosomal degradation impairment (Muñoz-Braceras et al., 2019), impaired autophagic degradation (Muñoz-Braceras et al., 2015), and abnormal calcium homeostasis (Pelzl et al., 2017). Furthermore, although VPS13A is not enriched in the synaptic compartment (Kurano et al., 2007; García-García et al., 2021), it is important for maintaining the neuronal optimal synaptic activity. Indeed, synaptic plasticity impairment and deficient glutamatergic and BDNF transmissions have been related to mouse corticostriatal VPS13A knockdown (García-García et al., 2023). In this line, enhanced neurite outgrowth and ramifications have been described in MSNs differentiated from hiPSC derived from fibroblast of ChAc (Stanslowsky et al., 2016), reinforcing the role of VPS13A in shaping neuronal dendritic morphology.

Interestingly, in HD, mHtt expression is also ubiquitous (Marques Sousa and Humbert, 2013). It accumulates in neurons as insoluble and hardly removable aggregates leading to synaptic abnormalities. Impairment of corticostriatal synaptic plasticity has been widely documented in HD mouse models and patients, with important glutamatergic and BDNF transmission disturbances in the molecular basis of these impairments (Canals et al., 2004; Del Toro et al., 2006). Furthermore, the accumulation of mHtt impairs a plethora of cell functions, including disruption of the ERK1/2 and the PKA signaling pathways (Saavedra et al., 2011; Tyebji et al., 2015). More interestingly mHtt also alters autophagy (Ravikumar et al., 2004), RNA splicing processes (Fernández-Nogales et al., 2016), and the rate of synthesis of selective proteins in the striatum (Creus-Muncunill et al., 2019), being these alterations at the basis of the molecular mechanisms of MSN dysfunction.

Considering all that, the involvement of VPS13A in the molecular mechanisms of HD pathophysiology cannot be discarded. The first step to approach this hypothesis is to depict the distribution of VPS13A in the HD brain, which should help to further understand its role in both basal ganglia synaptic plasticity and connectivity, and the ChAc neurodegenerative mechanisms. Thus, this study is focused on the analysis of the putative changes in VPS13A brain distribution induced by mHtt, especially in the striatum, cortex, hippocampus, and cerebellum.

## 2 Materials and methods

### 2.1 Human post-mortem nervous tissue

Human post-mortem tissue samples of the motor cortex and putamen were used to assess VPS13A concentration. Samples were collected at autopsy from individuals who had suffered a clinical history of HD (*n* = 3 female + 4 male, age: 54.4 (28–72) years; postmortem intervals of 4–18 h), and from non-HD controls (*n* = 4 female +2 male, age: 62.7 (39–81) years; postmortem intervals of 4–17 h; Table 1).

**Table 1:** Human post-mortem Huntington’s disease samples used in this study.

Patient	Pathological diagnosis	Gender	Age (years)	CAG repeats	Brain area
1	Control	Female	74	-	STR & CTX
2	Control	Female	60	-	STR & CTX
3	Control	Male	76	-	STR & CTX
4	Control	Female	71	-	STR & CTX
5	Control	Female	81	-	STR & CTX
6	Control	Male	39	-	STR & CTX
7	HD, Vonsattel grade 3	Male	55	48	STR & CTX
8	HD, Vonsattel grade 3	Male	85	40	STR & CTX
9	HD, Vonsattel grade 3	Female	65	45	STR
10	HD, Vonsattel grade 3	Female	72	42	STR & CTX
11	HD, Vonsattel grade 3	Male	53	45	STR & CTX
12	HD, Vonsattel grade 2	Female	28	62	STR & CTX
13	HD, Vonsattel grade 4	Male	60	43	STR & CTX

STR, Striatum (Putamen); CTX, Motor Cortex.

### 2.2 HD mouse model

Male and female R6/1 transgenic mice expressing the human exon-1 of mHtt containing 145 CAG repeats, and their corresponding wildtype (WT) littermates were obtained from Jackson Laboratory (Bar Harbor, ME, United States) and maintained in a B6CBA background. Animals were housed together in groups of mixed genotypes and kept under a 12:12 h light/dark cycle in a room at 19–22 °C and 40–60% humidity, with free access to food and water.

### 2.3 Mouse brain tissue sampling

Mice aged 20 weeks were used in the experiments. At this age, R6/1 mice show motor disturbances and synaptic plasticity impairment (Fernández-García et al., 2020; Kim et al., 2020). For immunohistochemical analysis, mice were anesthetized with a mixture of ketamine plus xylazine (100 + 10 mg/kg, i.p.) and transcardially perfused with ice-cooled 0.1 M PBS, followed by 4% paraformaldehyde (PFA). Then, brains were removed and fixed by immersion in 4% PFA at 4°C overnight. All PFA-fixed brains were cryoprotected with 30% sucrose in 0.1 M PBS and 0.02% sodium azide and frozen in dry ice-cooled isopentane. Specimens were stored at −80°C until sectioning. Sagittal serial sections were collected at 14 μm with a cryotome. For biochemical analysis, mice underwent euthanasia by cervical dislocation, and the brains were removed, dissected, and kept at −80°C.

### 2.4 Fluorescence *in situ* hybridization

A FISH procedure was performed using the RNAscope^®^ 2.5 High Definition–Red Assay kit (Advanced Cell Diagnostics, Newark, CA, United States), following the instructions of the manufacturer and as previously reported in García-García et al. (2021). The target probe for the mouse Vps13a gene (Probe-Mm-Vps13a-E61-E71-C2, Advanced Cell Diagnostics, Newark, CA, United States) was hybridized for 2 h at 40°C, followed by a series of signal amplification and washing steps. Hybridizations were performed in a HybEZTM Hybridization System (Advanced Cell Diagnostics, Newark, CA, United States). Negative controls were performed with a negative control probe (targeting the DapB gene from the *Bacillus subtilis* strain SMY) provided by the kit. Specific hybridization signals were detected by fluorescence, and RNA staining was identified as red dots.

Images of the Vps13a expression staining were obtained with an inverted microscope (Leica DMI6000 B, Thermo Fisher Scientific, Waltham, MA, United States). For qualitative visual analysis of the intensity of Vps13a mRNA labeling in the slices, digital images were processed using an 8-bit 16-color lookup table with ImageJ 1.51a (National Institutes of Health, Bethesda, MD, United States).

### 2.5 Quantitative real-time PCR

The aqueous phase containing total RNA was isolated from the different mouse brain regions using QIAzol (Qiagen, Hilden, Germany), following the protocol of the manufacturer. Total RNA isolation, reverse transcription of RNA and qRT-PCR were performed as already described (García-García et al., 2021). PrimeTime qPCR assays were used as recommended by the provider (assay code Mm.PT.56a.8500899, sequence NM_173028(1) for Vps13a, and assay code Mm.PT.39a.1 sequence NM_008084 for GAPDH; IDT technologies, United States). The expression level was determined using a standard curve and normalized to housekeeper Gapdh gene mRNA levels. The ΔΔCt method was used to analyze the data.

### 2.6 Western blot

Tissues were homogenized in lysis buffer and protein samples were resolved in SDS-PAGE and NuPAGE gels as already published (García-García et al., 2023). Immunoblots were probed with anti- VPS13A (1:1500, Cat: HPA021662, Sigma-Aldrich, St. Louis, MI, United States). Immunoreactive bands were visualized using the Western blotting Luminol Reagent (Santa Cruz Biotechnology, Dallas, TX, United States). Images were acquired using Chemidoc^™^ (Bio-Rad, Hercules, CA, United States) and quantified by a computer-assisted densitometer (ImageLab^™^, Bio-Rad, Hercules, CA, United States).

### 2.7 Statistical analysis

All experiments were blinded and randomized. All results are reported as mean ± SEM. Normal distribution of data was assumed when the Shapiro–Wilk test was positive for normality. Statistical analyses were performed using either a two-tailed Student’s t-test or one-way ANOVA followed by the Bonferroni test. Lineal regression models were generated to assess the relationship between the VPS13A levels in human brain samples and the HD Vonsattel’s grade or the number of CAG repeats. All statistic tests were performed on GraphPad Prism 9.0 (GraphPad Software, San Diego). Differential expression datasets from Microarray and RNA-seq analysis were identified in the Gene Expression Omnibus (GEO) repository from the National Center for Biotechnology Information (NCBI). The comparisons between control and HD conditions were performed with the GEO2R tool of the repository. In all analyses, values of *p* < 0.05 were considered statistically significant.

## 3 Results

**Figure 1:**
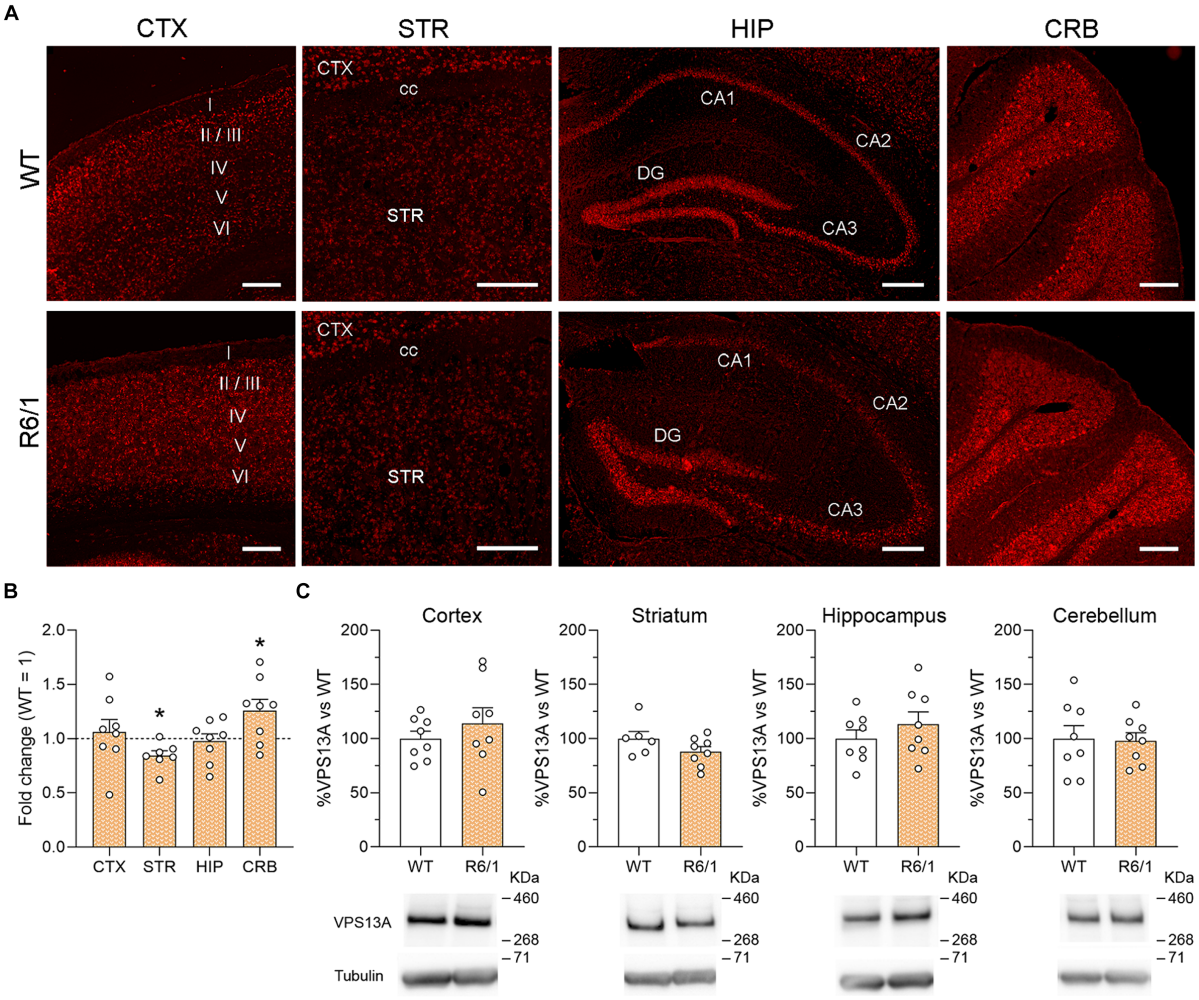
VPS13A mRNA and protein levels in representative brain regions of R6/1 mice. **(A)** Specific labeling of Vps13a mRNA in illustrative sagittal sections of striatum (STR), somatosensorial cortex (CTX), hippocampal formation (HIP) and cerebellum (CRB); cc, corpus callosum; STR, striatum; I, II, III, IV, V and VI are cortical layers; *n* = 3 WT and 3 R6/1 mice. Scale bar 250 μm. **(B)** Vps13a mRNA levels were analyzed by qRT-PCR in the striatum somatosensory cortex, striatum, hippocampus, and cerebellum. Values are expressed as mean ± SEM. Differences were analyzed by One-way ANOVA followed by the Bonferroni post-hoc test. *p < 0.05. Each point represents data from an individual mouse. *n* = 8 WT and 8 R6/1. **(C)** VPS13A protein levels were analyzed by western blot. Values are expressed as mean ± SEM. Differences were analyzed by Student’s *t*-test. Each point represents data from an individual mouse. *n* = 8 Ctrl and 8 R6/1 (CTX, HIP and CRB) and 6 WT and 8 R6/1 (STR).

To study the distribution of vps13a mRNA, we performed FISH on sagittal brain sections from WT and R6/1 mice at the age of 20 weeks. First, we focused on the brain regions of interest (Figure 1A) and we found different staining intensity profiles as previously published (García-García et al., 2021). We observed a staining enrichment in the cerebellum, notably in Purkinje cells and the granular layer; the pyramidal layers of the hippocampus and granular layer of dentate gyrus also presented high levels of staining. We found moderate staining in the somatosensory cortex and with wide expression along all cortical layers with a marked signal in layer V. Staining in the striatum was weak. We found no apparent differences in staining intensity between WT and R6/1 mice (Figure 1A).

We then analyzed the effects of mHtt on the VPS13A expression in the cortex, striatum, hippocampus, and cerebellum of 20-week-old R6/1 mice. After mRNA quantification by qRT-PCR, we found a significant 19% VPS13A expression decrease in the striatum and a 23% increase in the cerebellum of R6/1 mice when compared with WT animals (Figure 1B). However, these changes were not corroborated by western blot since we found no mHtt-induced changes in the VPS13A protein concentration in any of the four studied areas (Figure 1C).

**Figure 2:**
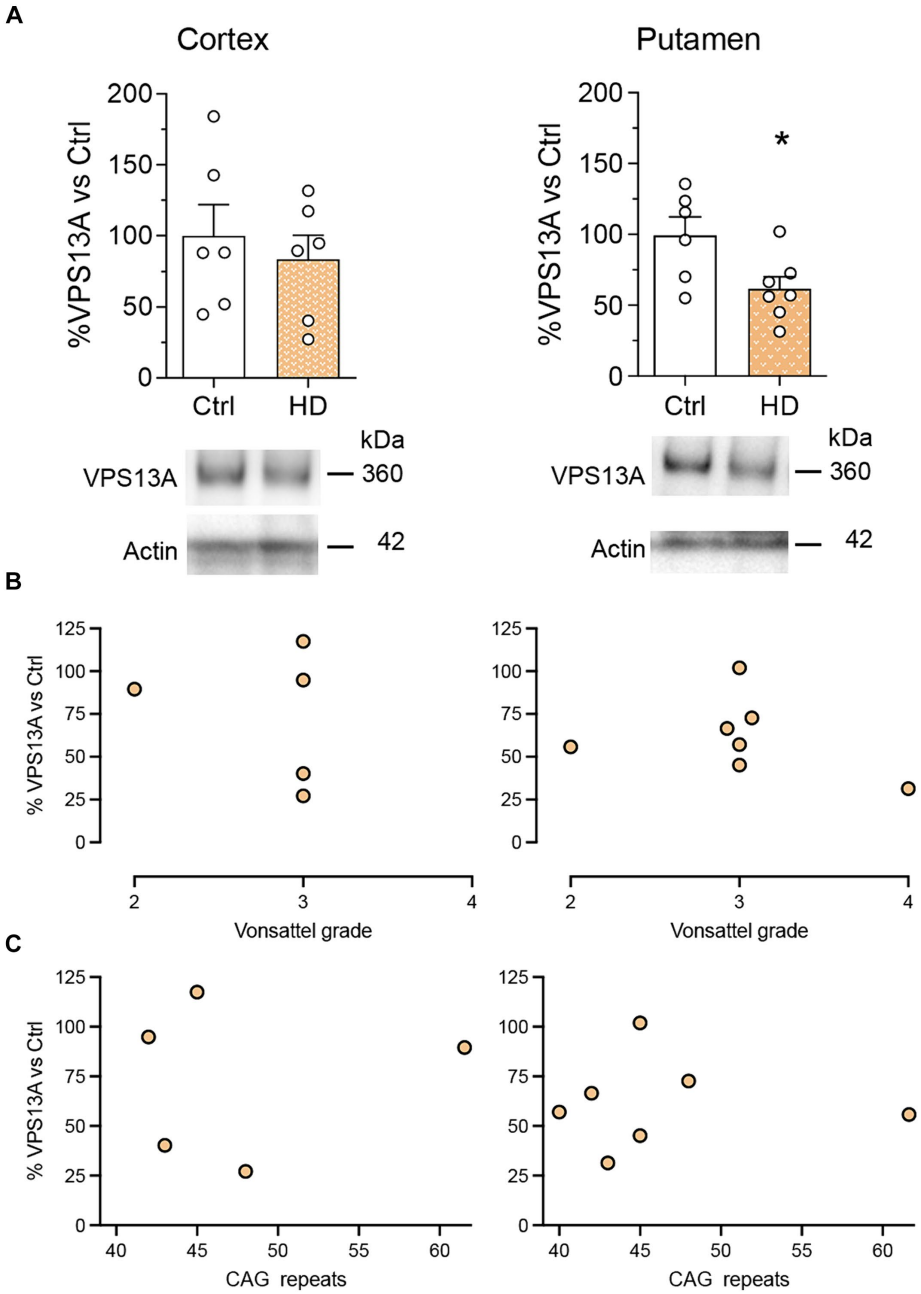
The levels of the VPS13A protein do not correlate with the HD condition. VPS13A protein levels were analyzed by western blot. **(A)** Representative immunoblots are shown. Values are expressed as mean ± SEM. Differences were analyzed by un-paired Student’s *t*-test. *p < 0.05. Dot plots show the lack of correlation of the VPS13A levels in the motor cortex and the putamen with either the neuropathological stage (Vonsalttel grade) **(B)** or with the number of CAG repeats in the Htt gene **(C)**. Each point corresponds to the value from an individual sample. *n* = 6 Ctrl and 6 HD (cortex) and 6 Ctrl and 7 HD (putamen).

We next analyzed whether this lack of significant VPS13A changes in symptomatic R6/1 mice was also present in the cerebral cortex and the putamen of HD patients. We found no significant changes in VPS13A levels in the motor cortex. However, we detected a significant 34% decrease in the putamen of HD patients compared with non-affected individuals by western blot (Figure 2A). The regression models showed no relationship between the levels of VPS13A protein with the HD Vonsattel’s grade in the motor cortex or the putamen (r^2^ = 0.005, *p* = 0.893 for the motor cortex and r^2^ = 0.098, *p* = 0.493 for the putamen; Figure 2B). Finally, we found no significant correlation between the levels of VPS13A and the number of CAG repeats in the motor cortex (r^2^ = 0.023, *p* = 0.772) or the putamen (r^2^ < 0.001, *p* = 0.994; Figure 2C).

To validate these results, we finally assessed the involvement of the VPS13A gene expression changes in HD in a series of differential-expression datasets published in the GEO. We compared the VPS13A expression in either, the motor cortex of HD-patients vs. controls (Lin et al., 2016), the whole blood of HD patients vs. controls (Hu et al., 2011), the iPSC-derived MSNs of HD vs. control patients (Chiu et al., 2015), the striatum of R6/2 mice vs. WT mice (Labbadia et al., 2011), and the conditionally immortalized HD STHdh^Q111/Q111^ striatal neuronal progenitor cell line vs. conditionally immortalized WT STHdh^Q7/Q7^ striatal neuronal progenitor cell line (Sadri-Vakili et al., 2007). According to the fold-change parameter of the VPS13A gene expression, with the *p*-value adjusted by the false discovery rate (Table 2), we found no significant HD effect in the VPS13A expression in any of the datasets analyzed.

**Table 2:** Differential expression analyses of changes in VPS13A expression in the human and mouse HD datasets.

GEO dataset	Organism	Samples	LogFCh	Adj. *p*
GSE79666	Human	Motor cortex	0.0206	0.9690
GSE24250	Human	Whole blood	0.2008	0.9980
GSE59051	Human	iPSC-derived neurons	0.4195	0.5970
GSE29681	Mouse (R6/2 model)	Striatum	0.0876	0.8058
GSE11358	Mouse	STHdh cells	0.2790	0.7410

LogFCh, logarithm of the fold change; Adj. *p*, adjusted *p*-value.

## 4 Discussion

With the evidence from the results obtained, we found that mHtt accumulation only subtly alters VPS13A, with some significant changes in the mRNA and protein concentration in the striatum but does not influence the tissue distribution. This indicates that VPS13A is a very stable protein with crucial functions in neuronal functioning. Previous results found that VPS13A neuronal content is stable over time and that its concentration is not modulated by the overactivation of cholinergic, dopaminergic, or glutamatergic systems (García-García et al., 2021). Altogether, these data suggest that VPS13A has a stable presence and role in neurons with a fine regulation of protein levels that maintains its steady role.

Our results validate previous observations of the heterogeneous VPS13A brain distribution (Kurano et al., 2007; García-García et al., 2021). Of the four analyzed regions, the striatum showed the lowest expression, also in the HD brain, although it is the most affected area in ChAc patients, while the cerebellum showed the highest expression, also in the R6/1 mouse model of HD. The fact that mHtt does not modify the brain distribution of VPS13A indicates a lack of interdependence of both proteins. Consequently, it suggests that their respective mutations might trigger convergent pathological mechanisms affecting the weak cellular properties of the MSNs. Although the protein tissue distribution is unaffected, the decrease in protein levels in the putamen of HD patients suggests that a direct interaction of VPS13A with mHtt cannot be discarded. As proposed for many other proteins (Wanker et al., 2019), a pathological interaction of VPS13A with mHtt may limit its functionality and biological functions, leading to VPS13A dysfunction, subsequent deficient phospholipid homeostasis (Miltenberger-Miltenyi et al., 2023), and mitochondrial function impairment.

In this regard, both VPS13A and mHtt have been involved in some common cellular processes. For example, data suggest the VPS13A involvement in the protein degradation machinery and autophagy in ChAc pathophysiology (Muñoz-Braceras et al., 2015; Lupo et al., 2016; Vonk et al., 2017), a process that is also altered in HD (Ravikumar et al., 2004). However, among all these common pathophysiological processes, mitochondrial dysfunction stands out. Recent studies showed that VPS13A is localized at sites where the endoplasmic reticulum (ER) and mitochondria are in close contact to enable lipid transfer required for mitochondria and lipid-droplet-related processes in cell lines (Yeshaw et al., 2019). Interestingly, structural and functional changes in the ER-mitochondria contact sites leading to mitochondrial dysfunction (Shirendeb et al., 2011; Cherubini et al., 2020), and aberrant lipid homeostasis or calcium signaling (Aditi et al., 2016; Pchitskaya et al., 2018; Tshilenge et al., 2023) have been reported to contribute to the specific degeneration of the striatum in HD (Browne, 2008).

Additionally, despite the low expression of VPS13A and mHtt in the striatum, both VPS13A reduction and mHtt accumulation have been associated with striatal synaptic plasticity impairment (Parievsky et al., 2017; García-García et al., 2023) and a reduction of signaling molecules important for synaptic functioning such as BDNF and CX3CL1 (Canals et al., 2004; Giralt et al., 2009; Kim et al., 2020; Azman and Zakaria, 2022; García-García et al., 2023). Strong evidence links these two proteins with altered neuronal communication and deficient long-term depression induction in the corticostriatal circuitry of HD (Baydyuk et al., 2011; Besusso et al., 2013; Kim et al., 2020) Thus, defining the role of VPS13A in striatal synaptic plasticity and MSN communication may constitute a key point to understanding the specific striatal vulnerability not only in ChAc, but also in HD.

Finally, the low number of HD-patient samples and transgenic animals herein analyzed constitutes a limitation of the study, as it may cause a loss of statistical power to detect differences in VPS3A expression and protein. Keeping this in mind, we conclude that VPS13A brain distribution is not substantially affected by mHtt. Therefore, the selective susceptibility of MSN in both ChAc and HD might be a consequence of disturbances in convergent cellular processes and circuitry alterations with divergent molecular mechanisms. Further experiments are necessary to evaluate the role of the VPS13A function in mediating these convergent mechanisms that determine MSN-specific vulnerability in basal ganglia disorders.

## Data availability statement

The raw data supporting the conclusions of this article will be made available by the authors, without undue reservation.

## Ethics statement

Human tissue samples were obtained from the Neurological Tissue Bank of the Biobanc- Hospital Clínic-Institut d’Investigacions Biomèdiques August Pi i Sunyer (IDIBAPS, Barcelona, Spain). The studies were conducted in accordance with the local legislation and institutional requirements. The participants provided their written informed consent to participate in this study. All animal procedures were conducted in accordance with the Spanish RD 53/2013 and European 2010/63/UE regulations for the care and use of laboratory animals and approved by the animal experimentation Ethics Committee of the Universitat de Barcelona (368/19) and Generalitat de Catalunya (11193).

## Author contributions

EG-G: Writing – original draft, Methodology, Investigation, Formal analysis. MC-C: Writing – review & editing, Investigation, Formal analysis. AC-M: Writing – review & editing, Investigation, Formal analysis. AR-L: Writing – review & editing, Investigation, Formal analysis. GB-S: Writing – review & editing, Formal analysis. EP-N: Writing – review & editing, Resources, Investigation. CM: Writing – review & editing, Resources, Funding acquisition. JA: Writing – review & editing, Validation, Resources, Project administration, Funding acquisition. MM: Writing – review & editing, Validation, Project administration, Funding acquisition, Conceptualization. MR: Writing – original draft, Supervision, Funding acquisition, Data curation, Conceptualization.
